# Aneurism of the Femoral Artery, Treated by Digital Compression—Cure

**Published:** 1874-12

**Authors:** James McCann

**Affiliations:** One of the Surgeons to Western Pennsylvania Hospital, Pittsburg, Pa.


ATLANTA

MEDICAL AND SURGICAL JOURNAL.

VOL. XII.] DECEMBER—1874. [NO. 9.

### Original Communications.

### ANEURISM OF THE FEMORAL ARTERY TREATED BY DIGITAL COMPRESSION—Cure in 11 Hours and 55 Minutes.

BY JAMES McCANN, M.D.,

*One of the Surgeons to Western Pennsylvania Hospital, Pittsburg, Pa.*

John Edwards, æt. 36, a stout, healthy looking Englishman, a "puddler" by occupation, was admitted to surgical ward of Western Pennsylvania Hospital, June 1, 1874, for an aneurism of the femoral artery.

He states that he has always been temperate and very healthy, never having had any serious illness in his life, except small-pox, when 14 years old. He had a venereal sore 17 years ago, which was not followed by any constitutional symptoms, although a chain of enlarged glands can be felt in each groin. His father died of consumption, and an uncle died of the same malady, but all the other members of his family are healthy.

The history which he gives of his aneurism is, that on the 23d of April last, while lifting a heavy box, he felt a sharp pain in his left thigh, as if a needle had been suddenly thrust into it. He experienced no further inconvenience from it at that time; but, during the ensuing night, he suffered pain in the limb, which he thought was Rheumatism. This pain continued to increase from that time, and was felt in the groin, around the hip, and down the inner side of the thigh and leg. Sometime during that week he discovered a small "lump" in his thigh, which "beat like his pulse." He continued to walk around, and finally obtained work, but was obliged to abandon it in a few days, and to seek relief in the hospital.

When admitted, a pulsating tumor, about the size of the half of a large orange, was visible in the left thigh, in the course of the femoral artery, just at the apex of Scarpa's triangle, and extending downward to about the commencement of Hunter's canal.

The pulsation in this tumor was very distinct; the peculiar thrill could be felt by the hand applied over it, and the ear readily detected a loud *bruit* synchronous with the radial pulse. Pressure applied to the artery on the cardiac side caused the tumor to grow smaller and softer, and arrested all pulsation in it. Removal of pressure was followed immediately by a return of the strong, heaving, "distensile" pulsation. The skin covering the tumor was unchanged, but the whole limb was larger than the opposite one, and the foot was somewhat œdematous.

He was ordered to be kept quiet in bed, and on light, nutritious food. A roller bandage was lightly applied to the limb, and the skin of the groin and inner part of the thigh frequently bathed with a solution of tannin in whisky, in order to toughen the skin and render it more tolerant to pressure. During the ensuing ten days no other treatment was instituted. The aneurism continued to increase in size steadily, and the pain in the limb became so great as to interfere with rest, and to require an opiate at night. The following measurements of the limbs were taken just before commencing compression:

Circumference of each thigh below trochanters, 18¾ inches.

Circumference of left thigh over aneurism, 19 inches.

Circumference of right thigh directly opposite aneurism, 18 inches.

Circumference of left thigh just above patella, 13¾ inches.

Circumference of right thigh just above patella, 13 inches.

Circumference of left leg at ankle, 8⅞ inches.

Circumference of right leg at ankle, 8⅛ inches.

Having obtained the assistance of six intelligent medical students, compression of the artery was commenced at 10:45 A.M., June 11, 1874.

The groin and inner side of thigh were smoothly shaven, and the limb enveloped in cotton wool from the toes to the aneurism. The students were divided into reliefs of two each, and were instructed to compress the artery with the thumb, applied over the vessel, just where it crosses the brim of the pelvis, and to use just sufficient force to arrest all pulsation in the aneurism. They were directed to alternate with each other at the end of every ten minutes; and, in alternating, the first relief was not to stop pressure until the second had the artery fairly under control. The skin of the groin was to be kept well dusted with powdered oxide of zinc.

A Signorim's tourniquet was now applied to the limb, and controlled the pulsation in the aneurism perfectly, but caused so much pain that it was removed at the expiration of fifteen minutes, and digital compression at once substituted for it. The following notes, (for which I am indebted to the residents, Drs. Mowry and Voigt,) taken at the bedside during the treatment, will indicate the progress of the case toward cure:

11:10 A.M.—Complains of pain in limb—Sulph. morphia, gr. $\frac{1}{4}$.

11:40 A.M.—Temperature right axilla, 100$\frac{1}{4}$; pulse 84.

12:10 P.M.—Complains of great pain in limb from point of pressure to toes.

12:46—Pain increasing—Sulph. morphia, gr. $\frac{1}{4}$.

1—Temperature axilla 99$\frac{1}{4}$; pulse 84. Still suffers pain.

2:12—Is sick at stomach, and complains of great pain in limb. Temperature axilla 100$\frac{1}{8}$; pulse 84—Sulph morphia, gr. $\frac{1}{4}$.

2:40—Passed f.℥ vss. urine, feebly acid, no albumen.

3:15—Temperature 100$\frac{1}{4}$; pulse 81; has slept for ten minutes. Consolidation has apparently commenced in tumor; it has diminished in size, and is firmer, and less pressure is required to control pulsation.

4—Still sick at stomach. Temperature 100$\frac{1}{4}$; pulse 88.

5 P.M.—Quiet, complains very little of pain. Temperature axilla 100$\frac{1}{2}$; toes 100; pulse 92.

6:15—Quiet; has short intervals of sleep; passed a small quantity of urine, feebly acid, no albumen. Says his foot begins to feel more natural; drank a glass of milk.

7—Temperature of toes of right foot 101. Drank a glass of milk.

7:50—Aneurism has diminished in size, and pulsation cannot be felt. Compression omitted for ten minutes and then re-applied. Circumference of thigh over aneurism 18 inches. Temperature 101$\frac{1}{4}$; pulse 96.

9:25—Temperature axilla 101$\frac{1}{3}$; left foot 100$\frac{1}{4}$; right foot 100$\frac{7}{8}$; pulse 84.

10:30—Temperature axilla 100$\frac{7}{8}$; left foot 99$\frac{3}{4}$; right foot 98$\frac{1}{4}$.

10:45—All compression stopped. There is now no pulsation in the tumor, which can be felt to have grown smaller and firmer. No pulsation can be detected in the posterior tibial artery, nor in the dorsal artery of the foot. The patient says that he is comfortable, and that he has suffered very little since 3 o'clock. A moderately firm compress was now applied over the tumor, and retained in position by strips of adhesive plaster, and the whole limb enveloped in a thick layer of cotton wool and light bandage.

11:45 P.M.—Complains of numbness in the leg—Morphia sulph., gr. $\frac{1}{4}$; tr. verat. virid., (Norwood) gtt. v.

June 12, 3 A.M.—Slept from 12 M. to 3 A.M.; pulse 80; temperature axilla $100\frac{1}{2}$; right foot $98\frac{1}{2}$; left foot $98\frac{3}{4}$—Tr. verat. virid., gttv.

6 A.M.—Slept from 4 to 5 o'clock. Pulse 84; temperature axilla $100\frac{1}{3}$; right foot $98\frac{1}{2}$; left foot $98\frac{3}{4}$—Tr. ver. vir., gtt. v.

11:10—Compress removed from tumor, and re-applied. No pulsation; temperature axilla $100\frac{1}{4}$—Tr. verat. virid., gtti. v.

1:50 P.M.—Pulse 82; temperature axilla $101\frac{1}{3}$; right foot $97\frac{3}{4}$; left foot $101\frac{1}{3}$.

4—Feeble pulsation detected in dorsal artery of foot.

5—Numbness of foot and leg, below ankle especially. Pinching back of foot and toes gives no pain. Pulse 77; temperature axilla $101\frac{1}{2}$; right foot $94\frac{2}{3}$; left foot $96\frac{3}{4}$—Tr. verat. virid., gtt. iv.

6—Bowels acted naturally.

7:30—Morphia sulph., gr. $\frac{1}{4}$.

8 P.M.—Removed bandage and compress. Slight movement perceptible in tumor by placing a slip of paper upon it. This evidently depends upon the impulse of the artery above, upon the solidified contents of the tumor. Patient complains of great tenderness in the groin and inner part of thigh at point where pressure was applied. Pulse 78; temperature axilla $100\frac{1}{4}$; right foot $99\frac{1}{4}$; left foot $98\frac{1}{4}$—Tr. verat. virid., gtt. v.

11—Pulse 80; temperature axilla $100\frac{1}{4}$; right foot $98\frac{1}{2}$; left foot $98\frac{1}{4}$—Tr. verat. virid., gtt. v.

13th, 2 A.M.—Sleeping soundly.

5—Pulse 70; temperature axilla $98\frac{1}{2}$; right foot $97\frac{5}{8}$; left foot 98—Tr. verat. virid., gtt. v.

8—Pulse 76; temperature axilla $99\frac{1}{2}$; right foot 94; left foot 96. No return of pulsation in tumor.

12 M.—Circumference of thigh over tumor $18\frac{1}{4}$ inches.

2 P.M.—Pulse 72—Tr. verat. virid., gtt. iv.

8—Pulse 82—Tr. verat. virid., gtt. iv.

9—Pulse 72; temperature axilla $100\frac{1}{2}$; right foot $98\frac{1}{2}$; left foot $98\frac{1}{2}$.

11—Pulse 74—Tr. verat. virid., gtt. iv.

14th, 1:20 A.M.—Morphia sulph., gr. $\frac{1}{4}$.

3 A.M.—Tr. verat. virid., gtt. iv.

14th, 6 A.M.—Tr. verat. virid., gtt iv.; sulph. morphia, gr. $\frac{1}{4}$. Pulse 76.

9 A.M.—Tr. verat. virid., gtt. iv.

12 M.—Tr. verat. virid., gtt. iv.

3 P.M.—Tr. verat. virid., gtt. iv. From this date he received no further medication.

15th—Condition unchanged; comfortable.

16th—Circumference of thigh over tumer $17\frac{1}{2}$ inches.

17th—Condition comfortable.

20th—Circumference of thigh over tumor 17 inches; circumference of right thigh $17\frac{3}{4}$ inches.

23d—Walked about ward for a short time on crutches.

26th—Pulse 76; temperature axilla $99\frac{1}{2}$; right foot $95\frac{1}{2}$; left foot 94. From this date improvement was steady and rapid; the circulation in the dorsal artery of the foot became more perfect, though it still remained feeble, as compared with that of the opposite limb, and the temperature became normal. All pain left the limb, and gained strength rapidly in it. He was discharged 26th of July, and on 1st of August began work as a laborer in a foundry.

September 26th, 1874—Patient reports to-day that he has been at work ever since 1st of August, at the heaviest labor he ever did in his life; that he has been in perfect health, and has "suffered no uneasiness in the leg whatever." The site of the aneurism can be felt as a somewhat elongated induration in the thigh, but is so small as not to be readily discovered unless sought for. There is no apparent difference in the size of the limbs, and the circulation in the foot seems natural.

The treatment of external aneurism has been wonderfully improved since the days of Anel and Hunter. Previous to their teachings the victim of aneurism had very little to hope for from any method of treatment except amputation; for the usual operation of opening the sac, turning out the clot, and endeavoring to secure both ends of the vessel, was very unsuccessful, and not unfrequently proved fatal on the spot. Death in all cases was to be apprehended, and, indeed, afforded the only prospect of relief where no operation could be attempted. The application of a ligature to the artery on the cardiac side of the tumor marked a new era in the surgery of aneurism; and, although the first attempts at cure by this method were unnecessarily severe and complicated, the success that followed them, when compared with the hazard, suffering, and almost uniform failure that attended all the old methods, at once attracted the attention of surgeons everywhere, and the operation, in the hands of its illustrious discoverer and his cotemporaries and pupils, soon became so simplified and perfected that there seemed to be nothing left that was desirable in the treatmeut of aneurism. A very remarkable degree of success attended this method in the hands of some surgeons. Syme, writing in the *Edinburgh Medical Journal*, May, 1866, stated that he had then applied the ligature to the femoral artery in thirty-five cases, and in "none of these has the operation proved unfortunate, with the exception of one, which terminated fatally from suppuration of the sac." Even this fatal case he was disposed to charge to "derangement of health, induced by a prolonged attempt to cause coagulation through pressure at the groin."

This great success is not the rule, however. Norris—*Contributions to Practical Surgery*—tabulates 204 cases of ligation of the femoral artery, including 13 of Syme's successful cases, with 50 deaths, or 24.5 per cent.; a result but little more favorable than ordinary amputation of the thigh.

Twelve of the patients were subjected to amputation, of whom only six recovered; and the question may well be asked, in how many of the remaining successful cases were the patients so perfectly restored to health as to be able to do any kind of labor, and in how short a time?

In three cases of which I have personal knowledge, the operations were performed by very skilful surgeons, and the aneurisms were cured, but the results were not at all satisfactory for many months afterward. The ligation of any large artery is an operation of considerable magnitude in itself, requiring skill and care in its performance. Syme states, (op. cit.) "I have seen a gush of dark-colored blood proclaim transfixion of the vein; I have seen, on dissection, a portion of this vessel included in the ligature; and I have also seen the external coat alone grazed, as it were, by the needle; but nevertheless excited to fatal inflammation."

So many difficulties and dangers attend the Hunterian operation that surgeons were naturally led to seek for a milder method of treating aneurisms. The fact that spontaneous cures occasionally occurred, and in cases considered irremediable, probably paved the way to this discovery; but it remained for the Dublin surgeons, Bellingham, Carte and Tufnelle, to perfect a plan of treatment by which an aneurism of a large artery can be cured in a short space of time, and without subjecting the patient to any risk or danger from an operation.* That method is compression of the artery on the cardiac side of the tumor; digital where possible, instrumental where digital is not available; a method which has now been applied with success in so many cases that its advantages over the ligature must be apparent. It causes very little suffering, rarely requiring an anæsthetic, (except in cases where rapid instrumental compression is used,) is followed by no shock, requires no wound of skin or loss of blood, and involves no danger to life. If it fails at first trial, it can be repeated again and again, and the ligature may be finally substituted for it, with better prospect of success, owing to the fact that the collateral circulation will be in some measure established by the previous treatment. It is applicable to all aneurisms, except those of the thoracic aorta and the arteria innominata; is most suitable for cases of false aneurism, growing with moderate rapidity, and to arterio-venous aneurism. It will fail most frequently in cases of tubular or fusiform aneurism.

In favorable cases, the time required to secure consolidation is often very short. A case is reported in the *Lancet*, April 24, 1874, in which this result was obtained by digital pressure in 90 minutes, in a traumatic femoral aneurism, occurring in a feeble man, 78 years old, and with diseased arteries; a case in which ligation would most likely have resulted fatally. In October, 1871, I saw, in Pennsylvania Hospital, Philadelphia, under care of Dr. R. J. Levis, a case of femoral aneurism situated just below Poupart's ligament, which had been cured by instrumental compression (under ether) in 5 hours and 33 minutes. I am not aware that this case was ever reported.

In regard to the length of time required in the present case, there is reason to believe that if pressure had been stopped at the end of 4 hours and 15 minutes (at 3:15 P.M.) the result would have been as perfect. No distinct pulsation was felt in the tumor after that hour, and the degree of force required to control the artery was so slight that one of the students was able to maintain pressure for one hour and twenty minutes continuously, and without fatigue.

In regard to the various methods of applying compression, it may be stated briefly that, where available, digital is much superior to any form of instrumental pressure, and for the simple reason that no instrument, however perfect, can ever be endowed with intelligence. What ever method is used, the amount of force expended should be just sufficient to completely arrest the current of blood in the aneurism, and no more. Any greater force causes unnecessary suffering to the patient, endangers sloughing of the skin, and does no good. The pressnre should, if possible, be applied to the vessel where it is most superficial, and can be most easily compressed against some bony prominence. In the foregoing case the pressure required to control pulsation in the tumor was slight when applied to the artery at the brim of the pelvis, but, when applied below, in the thigh, the amount of force required was much greater, and caused much suffering to the patient.

There is abundant reason to believe that hitherto the treatment of aneurism has been unnecessarily severe, and surgeons with the largest experience in the management of these cases are daily becoming more conservative. At a late meeting of the Royal Medical and Chirurgical Society of London, (see *Lancet*, March, 1874,) Mr. Joliffe Tufnelle asserted that "it was not necessary to resort to any operative means whatever in the treatment of external aneurism. He had cured it in twelve days, by low diet and rest. He had not selected his cases, nor had he refused any case. He had had fourteen successful cases out of eighteen." Such utterances, by so high an authority, certainly indicate a more conservative plan of treatment for aneurism than the ligature; and with such authority in favor of nothing but diet and rest, we should hesitate about resorting to an operation until we have exhausted every other means of cure. Much has been learned already in the treatment of aneurism, and doubtless much yet remains to be discovered before a perfect method is attained.

---

* The first case of aneurism treated successfully by digital compression alone occurred in the practice of Dr. Jonathan Knight, of New Haven, Connecticut, in 1848. The case was one of popliteal aneurism, and, all other methods of treatment having failed, it was determined to make a trial of digital compression before resorting to the ligature. Pulsation ceased in the tumor at the end of forty hours, and compression was stopped. The result was a perfect cure.—*Transactions American Medical Association*, 1848.

